# P-Hydroxyacetophenone Ameliorates Alcohol-Induced Steatosis and Oxidative Stress via the NF-κB Signaling Pathway in Zebrafish and Hepatocytes

**DOI:** 10.3389/fphar.2019.01594

**Published:** 2020-01-28

**Authors:** Sha Huang, Chuying Zhou, Ting Zeng, Yujia Li, Yuqi Lai, Chan Mo, Yuyao Chen, Shaohui Huang, Zhiping Lv, Lei Gao

**Affiliations:** ^1^ School of Traditional Chinese Medicine, Southern Medical University, Guangzhou, China; ^2^ The Key Laboratory of Molecular Biology, State Administration of Traditional Chinese Medicine, School of Traditional Chinese Medicine, Southern Medical University, Guangzhou, China

**Keywords:** p-HAP, alcoholic liver disease, zebrafish larvae, oxidative stress, apoptosis

## Abstract

Alcoholic liver disease (ALD), which is recognized as an important health problem worldwide, is a direct consequence of alcohol consumption, which can induce alcoholic fatty liver, alcoholic steatohepatitis, fibrosis and cirrhosis. P-Hydroxyacetophenone (p-HAP) is mainly used as a choleretic and hepatoprotective compound and has anti-hepatitis B, antioxidative and anti-inflammatory effects. However, no experimental report has focused on p-HAP in ALD, and the effect and mechanism of p-HAP in ALD remain unknown. In addition, there is no research on p-HAP in the treatment of ALD. The potential molecular mechanisms of p-HAP against acute alcoholic liver injury remain unknown. In this study, we aimed to investigate whether p-HAP alleviates ALD and to clarify the potential molecular mechanisms. Zebrafish larvae were soaked in 350 mmol/l ethanol for 32 h at 4 days post fertilization (dpf) and then treated with p-HAP for 48 h. We chose various outcome measures, such as liver histomorphological changes, antioxidation and antiapoptosis capability and expression of inflammation-related proteins, to elucidate the essential mechanism of p-HAP in the treatment of alcohol-induced liver damage. Subsequently, we applied pathological hematoxylin and eosin (H&E) staining, Nile red staining and oil red O staining to detect the histomorphological and lipid changes in liver tissues. We also used TUNEL staining, immunochemistry and Western blot analysis to reveal the changes in apoptosis- and inflammation-related proteins. In particular, we used a variety of fluorescent probes to detect the antioxidant capacity of p-HAP in live zebrafish larvae *in vivo*. In addition, we discovered that p-HAP treatment relieved alcoholic hepatic steatosis in a dose-dependent manner and that the 50 μM dose had the best therapeutic effect. Generally, this research indicated that p-HAP might reduce oxidative stress and cell apoptosis *in vivo* and *in vitro via* the NF-κB signaling pathway.

## Introduction

Alcoholic liver disease (ALD), which is caused by chronic or acute alcohol overuse, ranges from alcoholic fatty liver, alcoholic steatohepatitis, and fibrosis to cirrhosis (Axley et al., 2019). ALD threatens human health. “In 2016, worldwide alcohol‐attributable mortality was 38.8 per 100,000 people and 1,759 disability‐adjusted life‐years (DALYs) per 100,000 people” (Teschke, 2018; Axley et al., 2019). The underlying molecular mechanisms of ALD are associated with the hepatotoxicity of alcohol and its metabolites, oxidative stress and lipid peroxidation, cell apoptosis and autophagy, and other factors (Kong et al., 2019). The therapeutic guidelines for ALD are still dependent on avoiding alcohol abuse and ensuring only brief exposure to N-acetylcysteine, corticosteroids, pentoxifylline, anti-TNFα antibodies and liver transplantation (Ohashi et al., 2018; Crabb et al., 2019). In addition, numerous studies have indicated the potential of natural ingredients, for instance, flavonoids, β-carotene, resveratrol, and saponins, in targeted therapy for ALD (Hu et al., 2019; Tu et al., 2019). However, the effectiveness of treatments for ALD is not sufficient.

P-Hydroxyacetophenone (P-HAP), which naturally exists in *Artemisia capillaris Thunb.* and *Artemisia morrisonensis Hayata*, is used as a main hepatoprotective and choleretic compound and has anti-hepatitis B and some anti-inflammatory effects (Zhao et al., 2015; Ching-Wen et al., 2017). Moreover, further research found antioxidative and anti-inflammatory effects (Latif et al., 2019). However, there is insufficient evidence regarding the therapeutic effect and anti-steatosis ability of p-HAP in the context of liver diseases. The potential molecular mechanisms of p-HAP against acute alcoholic liver injury remain unclear, which restricts its clinical applications and keeps it from becoming a treatment for ALD. Consequently, there is an urgent need to investigate the pharmacological mechanisms of p-HAP in ALD and promote its clinical applications to save more lives.

Numerous studies in rodent models have substantively improved our understanding of ALD, but there are limitations (Zhang et al., 2016). In the past 20 years, zebrafish have become the main model system for studying the liver in vertebrates (Cox and Goessling, 2015). Similar to mammalian species, zebrafish have distinctive liver anatomical structures and cell structures, although the cell types in the liver are highly conserved (Goessling and Sadler, 2015). Furthermore, zebrafish produce large clutches of transparent embryos (> 200) in one mating. In addition, the expression and function of apolipoproteins in zebrafish and mammals are similar, which makes zebrafish a strong model of fatty liver disease for drug screening (Howarth et al., 2011). In previous studies (Lin et al., 2017; Zhou et al., 2017; Lai et al., 2018; Zhou et al., 2019), we screened several natural drugs with therapeutic effects on ALD based on high-throughput and convenient zebrafish experiments.

In this study, we adopted an *in vivo* zebrafish model and an *in vitro* cell model to study the potential molecular mechanisms and application value of p-HAP in ALD. We used the following indicators to evaluate the different outcomes of p-HAP treatment. H&E staining, oil red O staining and Nile red staining were used to observe the pathologic characteristics of the hepatic parenchyma tissue, especially the ectopic accumulation of lipids. In addition, different fluorescent probes were applied to detect ROS and GSH levels *in vivo*. For the first time, we discovered that p-HAP could repair alcoholic liver injury and elaborated the mechanisms by which p-HAP enhances liver repair, including the amelioration of hepatic steatosis and antioxidative and anti-apoptosis effects, after acute ethanol treatment, providing new ideas for the treatment of ALD.

## Materials and Methods

### Zebrafish Maintenance and Treatment

Wild-type (WT) and Tg (lfabp10α–EGFP) adults and larvae were maintained with a light: dark period of 14:10 h at 28°C. Embryos were gathered and developed in chorion water (0.5 mg/l methylene blue) for up to 5 days post fertilization (dpf) at 28.5°C. All zebrafish procedures were approved by the Institutional Animal Care and Use Committee of Southern Medical University.

Zebrafish larvae at 4 dpf were stochastically divided into two groups: the control group, which was raised in fish water, and the model group, which was kept in 350 mM ethanol for 32 h. P-HAP was soluble in dimethyl sulfoxide (DMSO) and diluted to 25 μM, 50 μM, and 100 μM with fish water. After exposure, the larvae were divided into 6 groups: the control group (fish water treatment), the 0.1% DMSO group (0.1% DMSO in fish water treatment), the model group (fish water treatment), and 3 therapy groups (25 μM, 50 μM, and 100 μM p-HAP treatment). The larvae were incubated in 6-well plates at a density of 30 larvae per well for 48 h.

### Cell Culture and Treatment

Human fetal hepatocyte cell line (LO_2)_ were cultured in DMEM containing 10% FBS, 100 units/ml streptomycin and 100 mg/ml penicillin (Gibco, Carlsbad, CA, USA) in a humidified atmosphere of 37°C, 95% air and 5% CO_2_. Cells were grown on six-well plates and were stochastically separated into a control group, model group and p-HAP treatment group. Previous studies have shown that curcumin pretreatment for 1 h before 100 mM ethanol exposure improved the result (Bao et al., 2010). The p-HAP treatment group was treated with p-HAP for 1 h before exposure to ethanol. Then, both the model group and the p-HAP treatment group were treated with 100 mM ethanol for 8 h. Cells were collected and proteins were extracted 8 h later.

### Histology

Zebrafish larvae were fixed in 4% PFA overnight at 4°C, routinely embedded in paraffin and sliced into 4 μm sections. Paraffin sections were dewaxed with xylene, dehydrated with different concentrations of ethanol, stained with hematoxylin and eosin, dehydrated, cleared, sealed, and finally filmed under a light microscope (Nikon Eclipse Ni-U; Nikon, Tokyo, Japan).

### Oil Red O Staining

Oil red O staining of zebrafish larvae and cryosections was performed as described previously (Zhou et al., 2019). Zebrafish larvae were fixed overnight in 4% PFA at 4°C, washed three times with PBS, and then incubated at room temperature with 20, 40, 80 and then 100% 1,2-propylene glycol (Sigma, USA) for 15 min each. Afterwards, larvae were incubated in oil red O solution (Sigma, USA) overnight at 4°C. The samples were rinsed in absolute propylene glycol for 60 min and washed with decreasing concentrations of 1,2-propylene glycol solution for approximately 30 min. Eventually, the larvae were washed with PBS and photographed under a bright-field dissecting microscope (Olympus U-HGLGPS; Tokyo, Japan).

For cell oil red O staining, the fixed cells were washed with PBS and stained with oil red O solution (0.7% oil red O diluted in isopropanol) for 8 min in the dark at 60°C, immediately decontaminated in 85% 1,2-propylene glycol for 5 min and washed with PBS 3 times. The results of oil red O staining were observed and photographed by optical microscopy (Nikon Eclipse Ni-U; Nikon, Tokyo, Japan).

### Nile Red Staining

The samples were fixed in 4% PFA overnight at 4°C and washed three times with PBS. After that, the cells were dyed with 0.5 µg/ml Nile red at room temperature for 10 min in the dark, washed three times with PBS and stained with a nuclear dye (DAPI, Solarbio Life Science, China) for 5 min in the dark at room temperature. Slides were imaged using an optical microscope (Nikon Eclipse Ni-U; Nikon, Tokyo, Japan).

### Immunochemistry

Paraffin sections (4 µm) were first dewaxed in xylene I, II, and III and then rehydrated in 100%, 95%, 90%, 80%, and 70% ethanol. Then, the samples were boiled in 1X sodium citrate, maintained at a subboiling temperature for 10 min to repair antigen and cooled to room temperature. Afterwards, endogenous peroxidase enzyme was inactivated using 3% H_2_O_2_ in methanol for 10 min in the dark at room temperature. Then, the sections were washed three times with PBS, sealed with 5% normal goat serum/1× PBS for 2 h at room temperature, and incubated with the primary antibody diluted in 2.5% normal goat serum/1× PBS overnight at 4°C. The primary antibody was a rabbit anti-NF-kB antibody (HuaBio, ER0815, 1:200 dilution). On the second day, the cells were washed three times with 1× PBS, incubated with a biotinylated goat anti-rabbit secondary antibody for 2 h at room temperature, washed three times with 1× PBS, and stained with DAB for 10 min. The reaction was stopped in ice water. Then, the samples were counterstained with hematoxylin, dehydrated, paraffinized and finally mounted and sealed with neutral gum. The dyed sections were photographed with an optical microscope (Nikon Eclipse Ni-U; Nikon, Tokyo, Japan).

### TUNEL Assay

The TUNEL reaction was used to detect hepatocyte apoptosis in liver tissue by using frozen sections and the In Situ Cell Death Detection kit (Roche). The cryosections were immersed in 0.01% Triton X-100 diluted in PBS for 10 min, washed with PBS, incubated with a 1:10 TUNEL working solution in a dark environment at 37°C for 1 h and washed three times with PBS. Then, DAPI was used to stain nuclei in the dark at room temperature for 5 min and then washed three times with PBS. The dyed cryosections were immediately photographed with an optical microscope (Nikon Eclipse Ni-U; Nikon, Tokyo, Japan).

### Superoxide and Glutathione Detection

The fluorescent probe dihydroethidium (DHE, Beyotime) is used as a fluorescent probe to detect O_2_- due to its reported relative specificity for this reactive oxygen species (ROS) (Gomes et al., 2005). A naphthylamide–sulfoxide-based fluorogenic probe (Na-8, Cat Number: FYRK-FP-01-003KY) is suitable for tracking fluctuations in endogenous GSH in living cells (Jiang et al., 2017). DHE and Na-8 were used separately to measure superoxide and glutathione in the following studies. After p-HAP treatment for 48 h, live larvae were immediately transferred into 24-well plates and incubated with a 10 μm solution at 28°C in the dark for 10 min. Then, the fluorescence distribution of DHE and Na-8 was visualized with a bright-field dissecting microscope (Nikon Eclipse Ni-U; Nikon, Tokyo, Japan).

### Western Blot Analysis

LO_2_ were collected and resuspended in RIPA lysis buffer cell lysis buffer (Sigma) containing a phosphatase inhibitor cocktail (Sigma) and a protease inhibitor cocktail (Sigma). A total of 30–50 μg protein was used for immunoblotting. An anti-caspase-9 antibody (rabbit, 1:1,000, Cell Signaling Technology), Alexa Fluor 568-conjugated goat anti-rabbit IgG (1:2,000, Invitrogen), and DAPI (Solarbio Life Science) were used to detect protein expression.

### Statistical Analysis

All statistical analyses were carried out by GraphPad Prism version 5.01 software and SPSS 20.0. The numerical results are shown as the mean ± standard deviation (SD). One-way ANOVA or an unpaired t-test was used for statistical analysis, and Tukey’s multiple comparison test was used for the appropriate experiments. P-values less than 0.05 were considered statistically significant.

## Results

### Toxicology of p-HAP in Zebrafish Larvae

**Figure 1:**
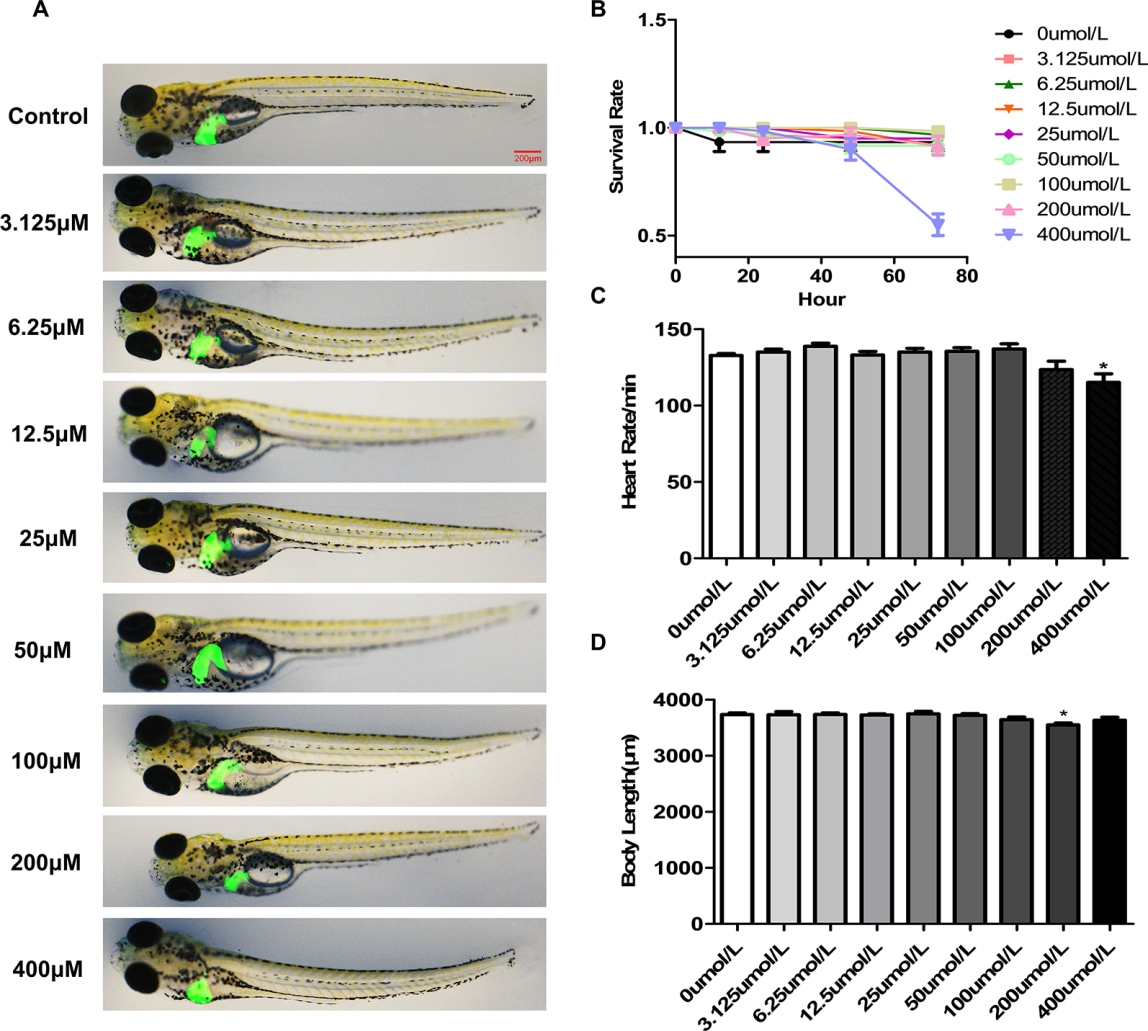
The toxicology of p-HAP in zebrafish larvae. **(A)** The developmental malformations in zebrafish larvae exposed to the indicated concentrations of p-HAP for 72 h. **(B)** Effects of different concentrations of p-HAP on the survival rate of zebrafish larvae. **(C)** Heart rate of zebrafish larvae exposed to different concentrations of p-HAP. **(D)** Body length of zebrafish larvae exposed to different concentrations of p-HAP. The data are displayed as the means ± SD, n = 10. (*P < 0.05 vs the control group).

We used the survival rate, the heart rate, morphological changes and body length to observe the toxicology of p-HAP in zebrafish larvae. Zebrafish larvae (4 dpf) were treated with p-HAP for 72 h. No morphological abnormalities in zebrafish larvae were observed at the tested concentrations of p-HAP (**Figure 1A**), indicating that p-HAP did have any toxic effects on the developmental stages of zebrafish. Notably, 400 µM p-HAP caused approximately 45% larval mortality after 72 h (**Figure 1B**). In the heart rate test, 400 µM p-HAP was toxic to zebrafish larvae (**Figure 1C**). In the body length test, compared with the control group, the 200 µM p-HAP group showed decreased body length (**Figure 1D**). Based on the results of the preliminary studies, we found that p-HAP was safe and had low toxicity, and we selected p-HAP concentrations of 25, 50, and 100 µM for further experimentation.

### P-HAP Attenuated Alcohol-Induced Hepatic Steatosis in an in *vivo* Zebrafish Model and an *in vitro* LO2 Cell Model

Previous studies have shown that exposure of 4 dpf zebrafish larvae to 350 mM ethanol for 32 h causes obvious hepatomegaly, vacuolization of the liver parenchyma and behavioral abnormalities and induces oxidative stress (Passeri et al., 2009; Quinlivan and Farber, 2017). Therefore, we exposed 4 dpf zebrafish larvae to 350 mM ethanol for 32 h as an alcoholic fatty liver model.

**Figure 2:**
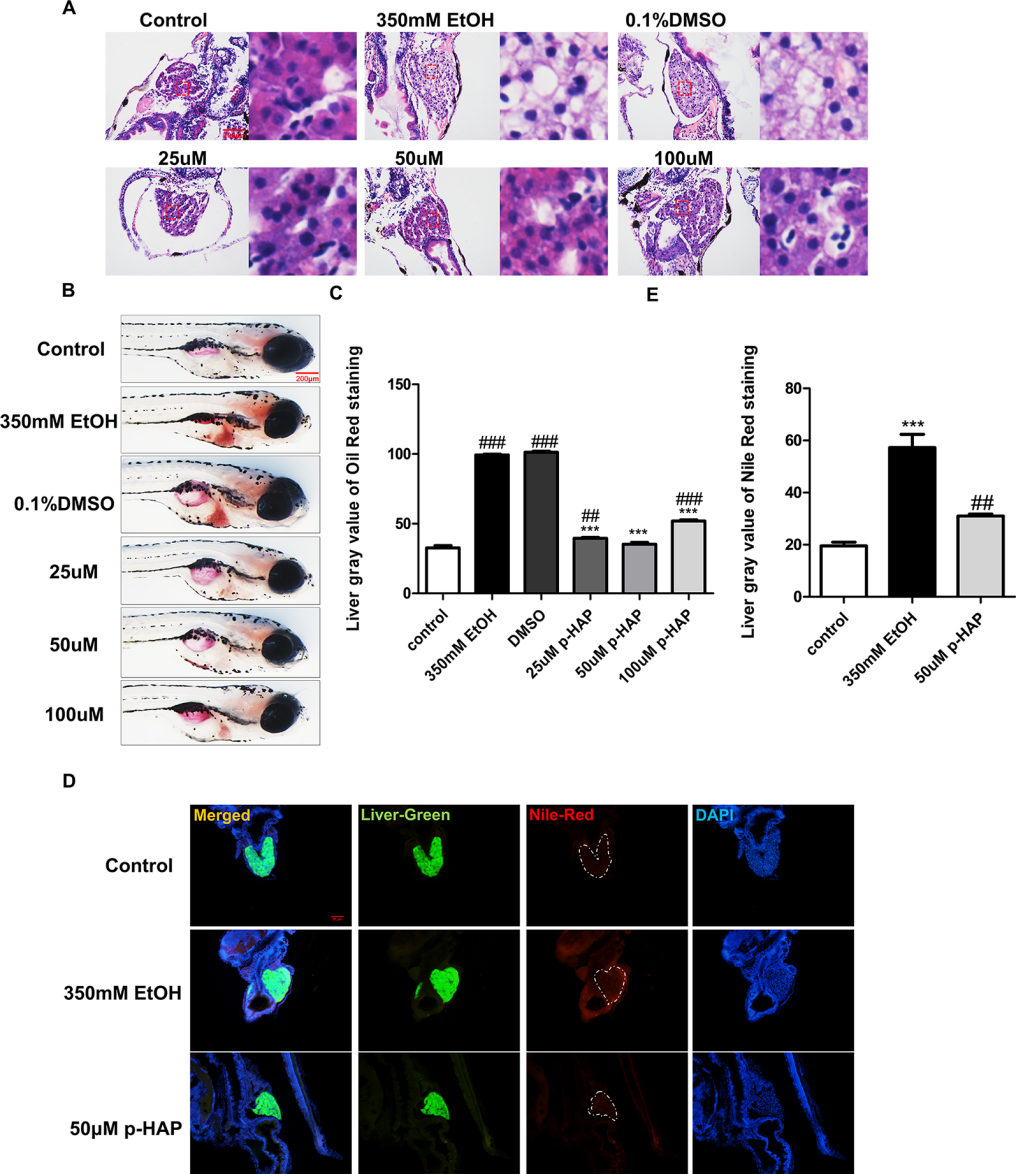
P-HAP attenuated hepatic steatosis induced by alcohol in zebrafish larvae. **(A)** H&E staining of the liver in zebrafish larva. Figures are magnified as 400×. **(B)** Lipid droplets in the whole-mount zebrafish liver were stained with oil red O after P-HAP treatment. Figures are magnified as 50×. **(C)** Oil red O staining of zebrafish larvae treated with 350 mM ethanol, 0.1% DMSO, and 25 μM, 50 μM, or 100 μM p-HAP and control zebrafish larvae were quantitatively analyzed. **(D)** The frozen liver sections from zebrafish larvae treated with 50 μM p-HAP were stained with Nile red. Figures are magnified as 200×. **(E)** Quantitative analysis of Nile red staining in zebrafish larvae. Data are expressed as the mean ± SD, n = 10 per group from two experiments using one-way analysis of variance (ANOVA) followed by Tukey’s multiple comparison test; ^##^P < 0.01, ^###^P < 0.001 vs control group, ***P < 0.001 vs model group.

Lipid peroxidation is a key feature of alcoholic liver injury. To evaluate whether lipids accumulated in the liver, hepatic tissues were evaluated by H&E, oil red O and Nile red staining. Histopathologic results indicated that treatment with p-HAP after ethanol exposure ameliorated hepatic steatosis and restored the liver tissue structures in zebrafish larvae (**Figure 2A**). Consistently, compared to those in the model group, the area and number of lipid drops detected by oil red O staining in the liver parenchyma of zebrafish larvae were significantly decreased after p-HAP treatment (**Figures 2B, C**). Based on the oil red O and H&E staining results, we found that 25-100µM p-HAP has therapeutic effect on alcohol liver injury, 50µM was the optimal concentration of p-HAP with anti-steatosis effects in the context of zebrafish alcoholic liver injury. Furthermore, as one of the most commonly used fluorescent dyes that binds to intracellular neutral lipids, Nile red (Greenspan and Fowler, 1985) was used and further confirmed that 50 μM p-HAP plays a protective role in alleviating lipid deposition in the liver of larvae after ethanol exposure (**Figures 2D, E**).

**Figure 3:**
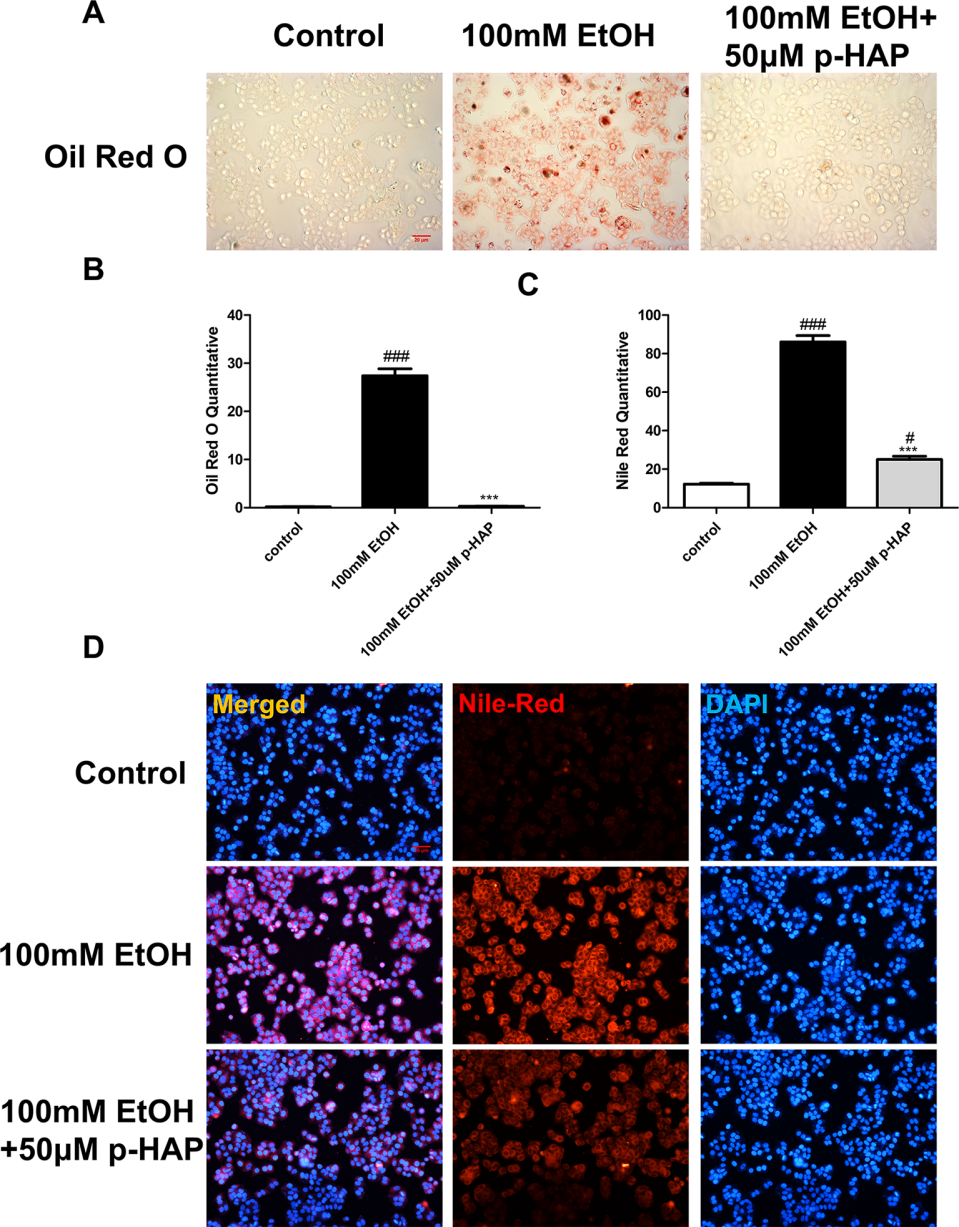
P-HAP reduced lipid accumulation inLO2 induced by alcohol. **(A)** Oil red O staining of LO_2_ slides. Figures are magnified as 100×. **(B)** Quantitative analysis of oil red O staining in LO_2_ treated with 100 mM ethanol, 50 μM p-HAP and the control. **(C)** Quantitative analysis of Nile red staining in LO_2_. The data are displayed as the means ± SDs (^#^P <0.05 vs the control group; *P <0.05 vs the 100 mM EtOH group). **(D)** Nile red staining of LO_2_. Figures are magnified as 100×. ^###^P < 0.001 vs the control group, ***P < 0.001 vs the 350 mM ethanol group.

On the other hand, we established *in vitro* alcoholic liver cell injury by exposing LO_2_ to 100 mM ethanol for 8 h according to previous studies, demonstrating that CYP2E1 protein levels increased in a time-dependent manner after exposure to 100 mM ethanol and peaked after 6 and 12 h (Liu et al., 2005). Consistent with the above zebrafish experiments, the *in vitro* ALD model was used to investigate the efficacy of p-HAP against hepatic steatosis. Oil red O staining indicated that 100 mM ethanol treatment for 8 h significantly induced intracellular lipid accumulation in LO_2_. However, intracellular lipid accumulation was reduced by p-HAP at a concentration of 50 μM (**Figures 3A, B**). Nile red staining was used to examine cellular lipid content, and the results revealed that 50 μM p-HAP significantly attenuated alcohol-induced lipid accumulation (**Figures 3C, D**).

These results indicated that p-HAP antagonized alcoholic liver injury by attenuating hepatic lipid accumulation; moreover, the effective concentrations of p-HAP were in the range of 25–100 μM, among which 50 μM was the most effective.

### P-HAP Protected Zebrafish Larvae Against Oxidative Stress After Alcohol Administration

**Figure 4:**
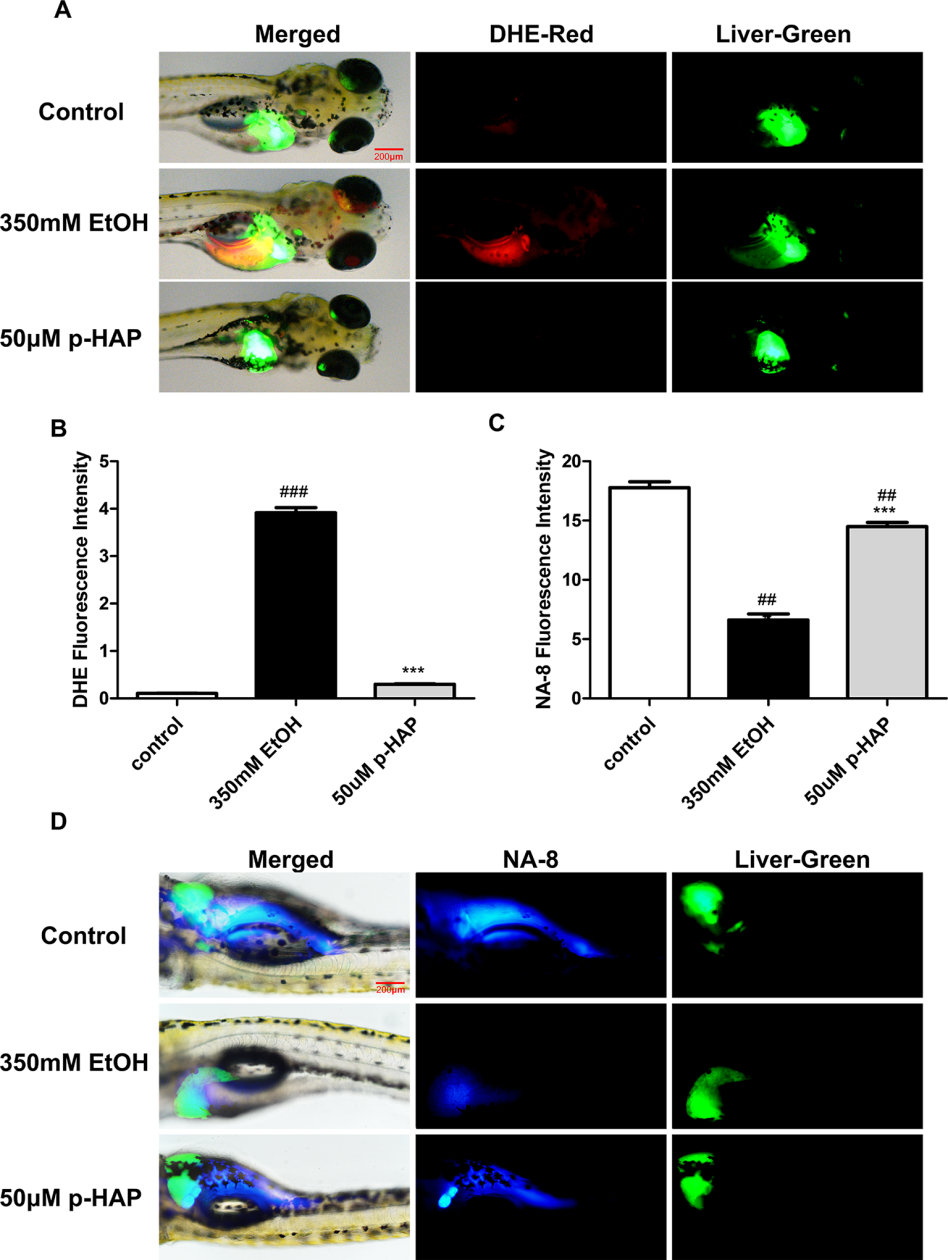
P-HAP protected zebrafish larvae from oxidative stress induced by alcohol consumption. **(A)** Fluorescence micrographs of ROS generation in the control zebrafish larvae and the zebrafish larvae treated with 350 mM ethanol or 50 μM p-HAP. Figures are magnified as 40×. **(B)** Quantification of the amounts and distribution of superoxide radicals according to fluorescence intensity. **(C)** Fluorescence micrographs of glutathione radical generation in zebrafish larvae. Figures are magnified as 40×. **(D)** Quantification of the amounts and distribution of glutathione anions according to fluorescence intensity. Data are presented as the mean ± SD. ^##^P < 0.01, ^###^P < 0.001 vs control group. ***P < 0.001 vs model group.

“High levels of reactive oxygen species (ROS) can lead to impairment of cell structure, biomolecules’ loss of function and cell death and are associated with liver diseases” (Miller et al., 2019). Oxidative stress induced by alcohol plays an important role in ALD (Tsedensodnom et al., 2013; Li et al., 2015). Moreover, decreased mitochondrial GSH is consistently observed in ALD and appears to be a key factor that contributes to ALD(Qu et al., 2019). To detect the distribution and levels of superoxide and glutathione radical distribution, we used liver-specific EGFP transgenic zebrafish [Tg(lfabp10α:EGFP)]. After the larvae were incubated with the DHE and Na-8 fluorescent probes for 10 min, we collected and photographed the larvae. DHE could be dehydrogenated by the action of superoxide anions in the cells after being taken up by living cells to produce ethidium. Ethidium (such as ethidium bromide) can combine with RNA or DNA to produce red fluorescence. When the level of superoxide anion in the cell is higher, more ethidium is produced, and the red fluorescence is stronger, and vice versa. The results indicated that the level of ROS in zebrafish livers increased notably, as the red fluorescence was stronger after ethanol exposure and decreased after treatment with 50 μM p-HAP (**Figures 4A, B**). The Na-8 fluorescent probe detected GSH in live cells, with an excitation wavelength of 405 nm and an emission wavelength of 498 nm. When the level of glutathione was increased, blue fluorescence was also increased. In our study, glutathione decreased in the ethanol-injured livers but increased after intervention with 50 μM p-HAP (**Figures 4C, D**).

These outcomes made it clear that oxidative stress plays a major role in ALD. p-HAP mitigated oxidative stress by reducing ROS and increasing GSH.

### P-HAP Protected Zebrafish Larvae Against Apoptosis and Inhibited NF-κb During Acute Alcoholic Injury

**Figure 5:**
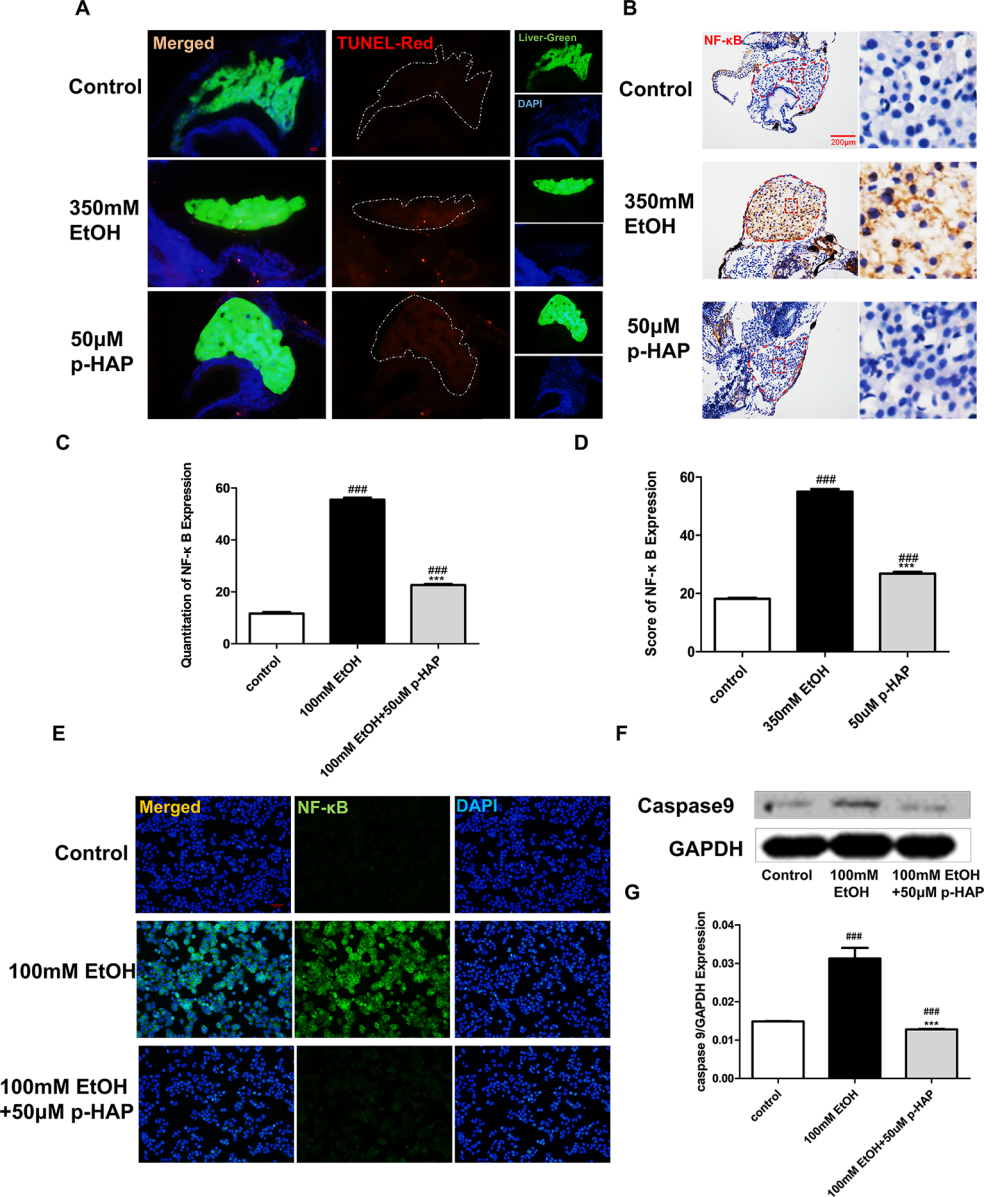
P-HAP Protected Zebrafish Larvae Against Apoptosis and Reduced Inflammation After Alcohol Administration. **(A)** *In situ* detection of cell apoptosis by TUNEL staining of paraffin liver sections in control zebrafish larvae and zebrafish larvae treated with 350 mM ethanol or 50 μM p-HAP. Apoptotic cells are indicated by white arrowheads. Figures are magnified as 400×. **(B)** NF-κB immunohistochemical staining of zebrafish larvae. Figures are magnified as 400×. **(C)** Quantitative analysis of NF-κB expression. **(D)** The amounts of NF-κB expression were quantified according to fluorescence intensity. **(E)** NF-κB immunofluorescence staining of LO_2_ slides. Figures are magnified as 200×. **(F)** Western blot analysis of caspase-9 expression in LO_2_ treated with 350 mM ethanol and 50 μM p-HAP. **(G)** Quantitative analysis of caspase-9 protein expression. Data are expressed as the mean ± SD. ^###^P < 0.0001 vs control group. ***P < 0.001 vs model group.

The transcription factor κ B (NF-κB) has been widely studied because of its significant role in the regulation of inflammatory genes and immune cell proliferation and apoptosis (Janssen-Heininger et al., 2000; Chen and Greene, 2004; Chen et al., 2019). It should be noted that NF-κB is the major regulator of oxidative stress (Chen and Greene, 2004). *In vivo* and *in vitro* results indicated that compared with the normal control condition, ethanol treatment significantly increased NF-κB protein expression, while 50 μM p-HAP treatment significantly decreased NF-κB protein expression (**Figures 5B, E**). Quantitative analysis of NF-κB protein expression was performed using ImageJ software (**Figures 5C, D**). Moreover, TNF-α protein expression was decreased in the 50 μM p-HAP treatment group while increased after ethanol treatment (**Supplementary Figure 1**). Furthermore, we conducted TUNEL assays to elucidate the antiapoptotic role of p-HAP in ALD. The administration of ethanol induced severe histological injury and apoptotic cell death in zebrafish larvae livers (**Figure 5A**). In addition, the caspase-9 axis is a frequently reported apoptotic pathway (Barman et al., 2016; Xiao et al., 2017). *In vitro*, compared with 100 mM ethanol treatment, 50 μM p-HAP treatment significantly downregulated the protein expression of caspase-9 (**Figures 5F, G**).

In conclusion, p-HAP can attenuate alcoholic liver injury by regulating NF-κB activation and apoptosis in hepatocytes and is likely an effective drug for the treatment and prevention of alcohol liver injury.

## Discussion

ALD threatens human health. The pathogenesis of ALD includes acetaldehyde-mediated toxicity, oxidative stress, hepatic steatosis, and an inflammatory response induced by cytokines and chemokines (Seitz et al., 2018). Alcohol abuse affects liver regulation of lipid metabolism and synthesis, leading to hepatocyte steatosis, which is the earliest histopathologic marker of ALD. In addition to lipid regulation abnormalities, ethanol-induced oxidative stress plays an important role in acute liver injury, as hepatocytes are the main cell types involved in ethanol metabolism (Garcia-Ruiz and Fernandez-Checa, 2018). When the concentration of ethanol is increased, ROS are generated by another alcohol-metabolizing enzyme, CYP2E1 (Leung and Nieto, 2013), which metabolizes ethanol to acetaldehyde, thus decreasing hepatic antioxidant defenses, especially GSH (Dai et al., 2003; Lu and Cederbaum, 2018). Besides, ROS also actives several downstream inflammatory pathways such as nuclear factor-κB(NF-κB). The current research on the mechanism of ALD is relatively sufficient, but effective treatments are few. The focus of this study was to find an active ingredient for ALD and clarify its mechanism.

**Figure 6:**
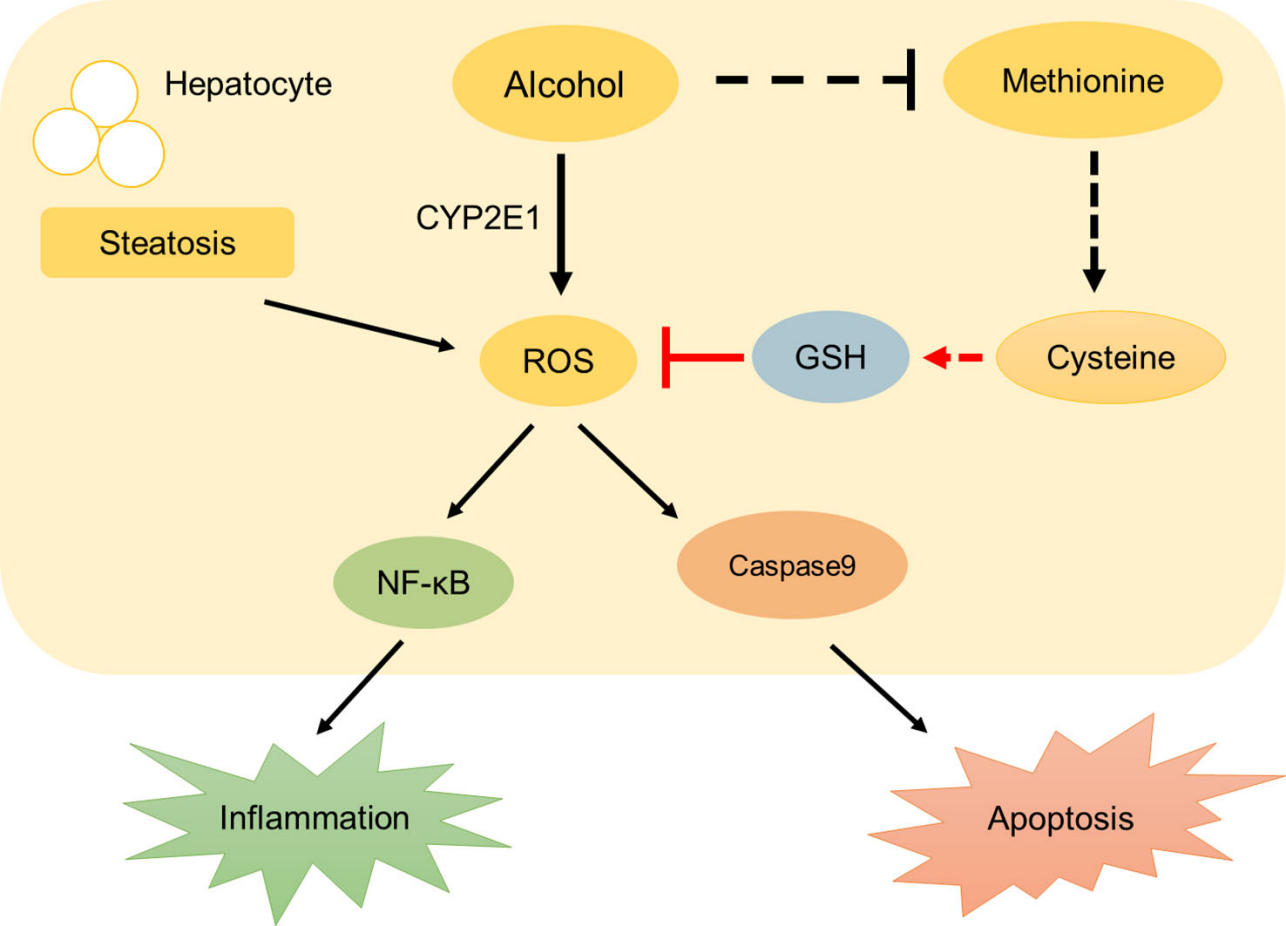
A schematic diagram describing the protective mechanisms of p-HAP against alcohol liver injury.

Our principal findings, derived from histological and molecular studies, are the following: First, liver steatosis and dyslipidemia occurs 32 h after acute alcohol exposure in zebrafish larvae. We find that p-HAP rescues alcoholic-induced liver injury and reduces lipid deposition in the liver, especially at a concentration of 50 μM p-HAP. Second, fluorescent probes are used to detect the distribution of ROS and GSH in zebrafish larvae. We prove that p-HAP had a specific antioxidative effect in ALD by reducing the level of ROS and increasing GSH. Third, oxidative stress activates the NF-κB pathway and stimulates caspase-9-related pathways, which causes hepatic parenchyma cell apoptosis and stimulates downstream inflammation-related proteins to cause liver inflammation (**Figure 6**). We demonstrate that p-HAP attenuates liver parenchyma apoptosis by inhibiting the NF-κB signaling pathway.

Commonly, oxidative stress is highly interrelated with multiple hepatic dysfunctions, including steatosis, lipotoxicity and inflammation (Zhang et al., 2019). Oxidative stress caused by ROS induces inflammation and cell apoptosis (Guo et al., 2019). The maintenance of intracellular ROS depends on functions of antioxidant systems, such as glutathione (GSH), glutathione peroxidase (GPx) and superoxide dismutase (SOD) (Guo et al., 2019). The liver has long been recognized as a reservoir for GSH, hepatocytes has the ability to synthesize a large amount of GSH, which is important not only for cellular redox balance but also for organismal cysteine balance (Stipanuk et al., 2006; Zhao et al., 2016). Hepatic ROS and GSH levels in zebrafish larvae are rarely reported. A previous study has reported a stable isotope dilution liquid chromatography/multiple reaction monitoring mass spectrometry (LC/MRM-MS) method to accurately quantify GSH and GSSG (Zhu et al., 2008). In our study, we use ROS and GSH fluorescent probes to detect the distribution of ROS and GSH in zebrafish larvae, which is intuitive and convenient.

Recent evidence demonstrated that apoptosis plays an important role in the pathogenesis of tissues and presents with morphological and biochemical features such as DNA fragmentation, cell contraction (Su et al., 2019). A key step in apoptotic signaling is the mitochondrial release of cytochrome c, which promotes the formation of apoptotic bodies and the activation of caspase 9, followed by caspase 3 (Riedl and Salvesen, 2007). NF-κB, as an important inflammatory transcription factor, is of great significance in regulating the signaling pathways related to pathological liver changes (Yakovleva et al., 2011). However, the role of NF-κB in apoptosis is still controversial. NF-κB activation usually results in the up-regulation of anti-apoptotic genes thereby providing cell survival mechanism to withstand the physiological stress that triggered the inflammatory response (Hoesel and Schmid, 2013). A previous study shows NF-κB mediated the up-regulation of Bcl-X_S_ and Bax in apoptosis (Shou et al., 2002). In our investigation, we discovered that the NF-κB pathway and caspase 9 were activated after alcohol exposure. P-HAP had protective effects on hepatocytes by inhibiting the expression of NF-κB and caspase 9.

In conclusion, the current study has successfully demonstrated the hepatoprotective effects of p-HAP on alcohol-induced acute liver injury and theorized that its mechanism might be partially related to its ability to regulate anti-inflammatory and apoptosis signaling pathways by inhibiting the expression of NF-κB and caspase-9 or by reducing ROS and increasing GSH content to resist oxidative stress. However, this study had some limitations. First, although we discovered that p-HAP could downregulate the level of ROS and upregulate the level of GSH in zebrafish with ALD, the specific regulatory mechanism remains unclear. As previous research showed, alcohol consumption inhibits the production of methionine, which promotes the production of cysteine, the raw material for GSH. Second, no related agonists and inhibitors and/or specific siRNA were used in the experimental model. The relationship between NF-κB and caspase-9 signal transduction remains to be elucidated, although we found that p-HAP could regulate the activation of NF-κB signaling. Third, even though we found that p-HAP could alleviate hepatic steatosis by reducing lipid accumulation, how it regulated lipid synthesis and metabolism has not been studied clearly. Based on the above shortcomings, our research group will further study and solve the above issues to determine the integral mechanism of p-HAP-mediated hepatoprotection against alcohol liver injury.

## Data Availability Statement

All datasets generated for this study are included in the article/**Supplementary Material**.

## Ethics Statement

The animal study was reviewed and approved by the Institutional Animal Care and Use Committee of Southern Medical University.

## Author Contributions

LG and ZL: research concept and design; design of the study. ShaHuang: participated in the main experiments. CZ, TZ, YujL, YuqL, and CM: organized the generation, collection, compilation, and interpretation of data. LG and ZL: participated in the drafting and revision of the manuscript. ShaoHuang, LG, and ZL: obtained funding. YC: provided scientific suggestions, research concept and design.

## Funding

This study was supported by the National Natural Science Foundation of China (81774170, 81603501, and 81673774), the Natural Science Foundation of Guangdong Province (2018B030306012), the Science and Technology Planning Project of Guangzhou City (201707010080), and the Scientific Research Initiative Program of Southern Medical University (CX2017N001).

## Conflict of Interest

The authors declare that the research was conducted in the absence of any commercial or financial relationships that could be construed as a potential conflict of interest.

## Abbreviations

p-HAP, p-hydroxyacetophenone; ALD, alcoholic liver disease; WT, wild-type; EGFP, enhanced green fluorescent protein; hpf, hours post fertilization; dpf, days post fertilization; DMEM, Dulbecco’s modified Eagle’s medium; FBS, fetal bovine serum; PBS, phosphate-buffered saline; RIPA, radioimmunoprecipitation; PFA, paraformaldehyde; DMSO, dimethyl sulfoxide; OCT, optimal cutting tissue; H&E, hematoxylin and eosin; IF, immunofluorescence; IHC, immunohistochemistry; LO_2_, human fetal hepatocyte cell line; ROS, reactive oxygen species; DHE, dihydroethidium; GPX4, glutathione peroxidase 4; GSH, glutathione; NF-κB, nuclear factor kappa-B; ANOVA, analysis of variance.

## Supplementary Material

SUPPLEMENTARY FIGURE 1 TNF-α immunohistochemical staining of zebrafish larvae.
