# Supplementary figures and images for: P-Hydroxyacetophenone Ameliorates Alcohol-Induced Steatosis and Oxidative Stress via the NF-κB Signaling Pathway in Zebrafish and Hepatocytes
Source: Front Pharmacol. 2020 Jan 28;10:1594. doi: 10.3389/fphar.2019.01594 (PMC6997130; doi:10.3389/fphar.2019.01594)

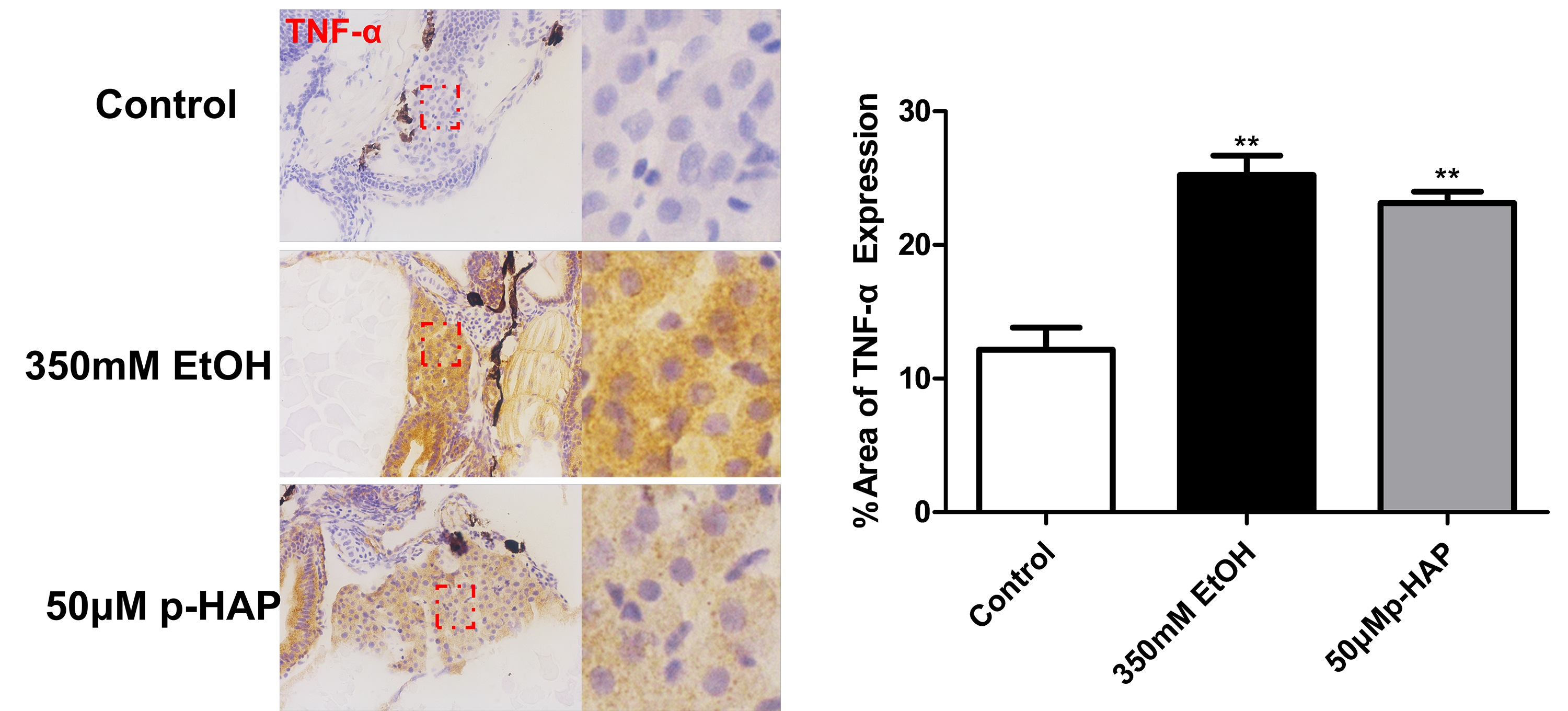

Supplement: SUPPLEMENTARY FIGURE 1 — TNF-α immunohistochemical staining of zebrafish larvae. [file Image_1.tif]
